# Supplementary material for: Learning curve and surgical outcome of robotic assisted colorectal surgery with ERAS program
Source: Sci Rep. 2022 Nov 29;12:20566. doi: 10.1038/s41598-022-24665-w (PMC9709162; doi:10.1038/s41598-022-24665-w)
Supplement: Supplementary file 2 — Supplementary Information 2. [file 41598_2022_24665_MOESM2_ESM.zip › RAL Raw data-Ñ[▒K/111/HS_ñw╝╨░O▒Kñσ.pdf]

正式摘要(電子簽章：0000008226198733)

W52 / W52 ☐ 英文標題(注意:無英文姓名-請補病人基本檔!) ☐ 【讀取】憑證

臺中榮民總醫院

出院病歷摘要

轉入號： 轉出號：

[redacted]

(8)流水編號	
(9)入院日期	110/09/08 (2021)
(10)轉科日期	
(11)出院日期	110/09/24 (2021) 住院天數計 16 日

診斷

- 入院：1. Adenocarcinoma of rectum
- 出院：1. Adenocarcinoma of rectum, s/p robotic low anterior resection + loop ileostomy on 2021/09/09, pT2N0
  2. Postoerative ileus, improved under conservative treatment

主訴

- blood-tinged stool for one year

病史

- This 32-year-old man denied past history was suffered from blood-tinged stool for one year. He had visited 鹿港基督教醫院 for upper & lower GI scopy survey and rectal adenocarcinoma had been proved. He came to our hospital for second opinion on 110/08/24. As above problems, he was admitted for surgery.

體檢發現

-
  - General appearance :
    - easy looking , conscious clear , without cardiopulmonary distress
    - Vital signs自動帶入date 體溫(BT) 脈搏(P) 呼吸(R) 血壓(BP) 身高(BH) 體重(BW) BMI
      20210908 36.3℃ 77次/分 18次/分 149/87mmHg 174.3cm 69.7kg 22.9
    - Vital signs: B.P. 149/87 mmHg , P.R. 77 /min , R.R. 18 /min , B.T. 36.3 ℃
  - HEENT :
    - Eyes: no anemic conjunctiva, no icteric sclera, normal EOM
    - ENT: no otorrhea, no rhinorrhea, no erythematous mucosa, no injected tonsil, no deformity
  - Neck: supple, freely movable, trachea midline,no deformity, no engorged jugular vein , no palpable thyroid or lymph node enlargement
  - Chest:
    - Inspection: Grossly normal, symmetric and fully expansion, no accessory muscle used, no operation scar, no spider angiomata, no gynecomastia
    - Palpation: no palpable mass or nodule, no subcutaneous emphysema, no tenderness
    - Ausculation: clear breathing sound
  - Heart:
    - Percussion: normal heart size
    - Ausculation: regular heart beat, normal S1, S2, no gallop, no murmur
  - Abdomen:
    - Inspection: in
      - flat contour, symetrically, no scar, no stria, no dilated veins, no rashes
    - Ausculation: normoactive bowel sound, no abdominal bruits
    - palpation: soft, no muscle guarding or rigidity, no local tenderness, no rebounding pain, no palpable mass, no palpable pulsation
    - Percussion: no shifting dulllness, no hepatomegaly, no splenomagaly
  -

Peripheral pulsation :	CA	BA	RA	FA	PA	PTA	DPA
Right side :	++	++	++	++	++	++	++/+++
Left side :	++	++	++	++	++	++	++/+++

  - Genito-anus: no tenderness or palpable mass lesion or rectal shelf, normal external genitalia, normal anal tone, brown stool

CRS CONDITION

- CEA: 1.57 ng/ml
-
  - Lung

手術日期及方法

- 手術日期及方法20210909 1. Robotic (XI) assisted low anterior resection
  2. Intraoperative colonoscopy : (-).
  3. High ligation (+) : At IMA root (Via robotic clips). IMV was ligated via endoclip.
  4. Take down from the splenic flexure : (-)
  5. Robotic EndoGIA (45-4.3) X2 + EEA (33mm) + OpenGIA (80-3.8) X1 were applied to complete the side-to-end anastomosis
  6. Proximal cut end: > 10 cm, distal cut end: 1.5 cm
  7. No drainage tube was inserted
  8. ADHESION BARRIER FILM was placed
  9. Loop ileostomy (About 15~20cm from IC valve) was done over RLQ of abdomen
  # Notice : While firing the second staple of the robotic endoGIA, malfunction was detected. Special device was applied to release the GIA. A new instrument was used instead, under the safety concern. The third robotic endoGIA was then fired successfully.
- 手術結果及發現：
  - 1. A 4 x 4 cm tumor at lower rectum. (DRE : Finger tip could reach the lower edge of the tumor) 2. No other gross tumor nor polyps was found 3. There was no obvious visible liver metastasis, no visible peritoneal seeding 4. No obvious ascites was noted 5. Bilateral doughnuts : intact ; Air leak test: Done and no air leak was found

住院治療經過

- After admission, he underwent ERAS-based preoperative prepare, then robotic-assisted low anterior resection + loop ileostomy smoothly on 2021/09/09. After surgery, he had regained conscious and kept ERAS-base postoperative care. However, he was suffered from moderate abdominal pain, he also complained right lower limb pain and numbness, especially during using PCA control therefore Anesthesia doctor re-inserted epidural PCA on 09/10. He still complained about abdominal fullness and nausea, KUB (9/12) showed intestine gaseous loop dilatation. We applied NG decompression and much fluid 800ml drainage was noted. Foley was removed at POD-2, but he was suffered from urine retention and re-on Foley at the same night. During this week (9/13-9/19). He was suffered from full abdominal with tenderness, KUB(9/14) showed much gaseous distension of small bowel with nearly absent bowel gas in colon. We inserted anal tube into ileostoma on 09/14, then there was much ileostoma output through anal tube and he got markedly improvement on distented abdomen. NG tube was removed. We kept PPN support on 09/16 (POD-7). During this week (9/20-9/23) He started to try liquid diet on 09/20, semi-liquid diet on 09/22, soft diet and removed anal tube on 09/23. Under his relative stable condition, he was discharged on 09/24.

合併症

- Ileus

一般檢查

- 累積報告：

DATE	TIME	ALB	TP	BIL.T	BIL.D	ALKP	AST	ALT	LDH	AMY	LIPASE	NH3	Ferritin	UA
1100909	2226	3.7		0.71	0.16		17	21						

1100913	1458	3.4		0.5	0.2		18	12						
1100915	1110			0.4	0.1		15	11						
1100917	0857	4.0												
1100920	0853	3.9		0.53			28	38						

DATE	TIME	WBC	RBC	HGB	HCT	MCV	PLT	BLST	NEUT	BAND	SEG	LYM	MON	EOS	BAS
1100909	2226	17630	4.67	12.8	40.1	85.9	269		91.8			6.0	2.0	0.0	0.2
1100913	1459	4160	4.57	12.4	38.2	83.6	215			6.0	65.0	16.0	13.0	0.0	0.0
1100915	1118	5030		12.9	38.5		189			2.0	70.0	17.0	11.0	0.0	0.0
1100917	0918	10290		14.7	44.9		253			1.0	69.0	24.0	4.0	0.0	0.0
1100920	0933	9330		14.0			321				72.0	19.0	7.0	1.0	0.0

DATE	TIME	NA	K	CL	BUN	CREAT	eGFR	CA	Mg	P	CRP	ESR	PCT	D-Dimer	LACTATE
1100909	2226	143	3.8	105	11	0.94	98.85	8.9							43.5
1100913	1458	140	3.4		12	0.72	134.46	8.0							
1100913	2005								2.0	1.4					
1100915	1053														7.0
1100915	1110	138	2.9		8	0.72	134.46	8.1							
1100917	0849														7.4
1100917	0857	139	3.6		11	0.83	114.12	9.3	2.5	4.3					
1100920	0853	136	4.4		12	0.79									

DATE	TIME	GLUCOSE	HbA1C	CHOL	TG	HDL	LDL	KETONE	CK	CKMB	TRO-T	PT-P	APTT-P	NT-pro BNP
1100913	2005			132	54									

- 放射線報告 :檢驗項目 **:ABDOMEN (KUB),SUPINE** 報告日期 **:1100923**

  主　訴：
  Result:N
  Finding:
  > Increased bowel gas with mild dilated small bowel loops, obstruction or ileus should be considered. Suggest clinical correlation.
  > The bony structure seems to be intact.
  > S/P ostomy over RLQ of abdomen.
  > Obscured margin of bilateral psoas muscle and kidney shadows due to bowel gas
  > Surgical autosuture retention over pelvic region
  Impression:
  As above description.

  檢驗項目 **:CT ABDOMEN-SURVEY** 報告日期 **:1100923**
  主　訴：
  Result:Y
  Finding:
  CT scan of abdomen with IV contrast enhancement:
  > History of rectal cancer S/P LAR
  > No significant space occupying lesion or wall thickening over anastomosis site in pelvis
  > S/P enterostomy over RLQ of abdomen with a drainage tube inserted via ostomy into pelvis, causing obstruction of ileum with dilatation of proximal ileum and jejunum
  > Wall thickening and enhancement of proximal ileum and jejunum, r/o enteritis
  > Edematous change of submucosal layer of ascending colon
  > Mild stranding over mid-abdominal wall (Axi:45-55), may due to post-operation change. Suggest follow up.
  > Mild fluid accumulation over pelvis
  > A 0.7cm fat-contained nodule over upper pole of right kidney (Axi:26), r/o renal AML
  > The pancreas, spleen, gallbladder, left kidney and visible lung fields are without specific abnormality
  > No significant lymphadenopathy at retroperitoneum and bil. pelvic region
  Impression:
  1. S/P enterostomy over RLQ of abdomen with a drainage tube inserted via ostomy into pelvis, causing obstruction of ileum with dilatation of proximal ileum and jejunum
  2. Wall thickening and enhancement of proximal ileum and jejunum, r/o enteritis

---

特殊檢查

病理報告

- 活體組織切片病理報告　手術日期：**110/09/09**　病理號：**S1028427**

  診斷醫師:李欣倪 報告日期:110/09/15
  Pathologic diagnosis:
  1. Rectum, robotic low anterior resection --- Adenocarcinoma, moderately differentiated, arising in a tubulovillous adenoma. Tumor invaded to muscularis propria. The bilateral cut ends and perirectal lymph nodes (0/12) are free of tumor.
  2. IMA root, dissection --- Unremarkable (0/1).
  3. Rectum, cut end, distal, resection --- Chronic inflammation.
  Ancillary study for diagnosis:
  IHC stains for PMS2 and MSH6 are done on section C. Both above markers are preserved.
  Prognostic and predictive factor:
  1. Tumor size: 3.1 x 3 cm.
  2. Depth of invasion: Muscularis propria.
  3. Histologic grade: G2: Moderately differentiated.
  4. Angiolymphatic invasion: Not identified.
  5. Perineural invasion: Not identified.
  6. Tumor deposits (discontinuous extramural extension): Not identified.
  7. Macroscopic tumor perforation: Not identified.
  8. Surgical Margins: a) Proximal Margin: Uninvolved by invasive carcinoma, distance of tumor from margin: 15 cm. b) Distal Margin: Uninvolved by invasive carcinoma, distance of tumor from margin: 2.1 cm. c) Radial or Mesenteric Margin: Uninvolved by invasive carcinoma, distance of tumor from margin: 2.8 cm.
  9. Lymph node status: Uninvolved (0/13).
  10. Extranodal involvement: Not identified.
  11. Tumor regression grading S/P CCRT: No known presurgical therapy.
  12. Type of polyp in which invasive carcinoma arose: Tubulovillous adenoma.
  13. Additional pathologic findings: Not identified.
  14. Pathological TNM Stage: pT2N0 (According to the eighth edition, American Joint Committee on Cancer Staging Guidelines for Tumors).
  15. TNM descriptors: Not applicable.
  Gross description:
  The specimen consists of 1) a segment of colon, 21 cm in length and 4.5 cm in proximal cut end circumference, 8 cm in distal circumference, and perirectal soft tissue. A gray tan polypoid tumor, 3.1x3 cm, is noted, 2.1 cm to the distal cut end. On cross section, the tumor invades to the muscular layer with 2.8 cm to the radial margin. The both cut ends are free of tumor grossly. 2) IMA root, a piece of tan yellow soft tissue, 2.5x1.3x0.6 cm. 3) distal cut end, a piece of tan mucosa-covered soft tissue, 2.6x1.3x0.8 cm.

- Representative part for section: A) proximal cut end B-E) tumor and radial margin F) specimen 2 G) specimen 3 H-J) lymph nodes.
  Note:
  1. The interpretation of MMR panel and recommended studies are as below: (1) No loss of nuclear expression of MMR proteins: low probability of microsatellite instability-high (MSI-H) . (2) Loss of nuclear expression of MLH1 and PMS2: testing for methylation of the MLH1 promoter and/or mutation of BRAF is indicated (the presence of a BRAF V600E mutation and/or MLH1 methylation suggests that the tumor is sporadic and germline evaluation is probably not indicated; absence of both MLH1 methylation and of BRAF V600E mutation suggests the possibility of Lynch syndrome, and sequencing and/or large deletion/duplication testing of germline MLH1 may be indicated). (3) Loss of nuclear expression of MSH2 and MSH6: high probability of Lynch syndrome (sequencing and/or large deletion/duplication testing of germline MSH2 may be indicated, and, if negative, sequencing and/or large deletion/duplication testing of germline MSH6 may be indicated). (4) Loss of nuclear expression of MSH6 only: high probability of Lynch syndrome (sequencing and/or large deletion/duplication testing of germline MSH6 may be indicated). (5) Loss of nuclear expression of PMS2 only: high probability of Lynch syndrome (sequencing and/or large deletion/duplication testing of germline PMS2 may be indicated). (6) Please note: There are exceptions to the above IHC interpretations. These results should not be considered in isolation, and clinical correlation with genetic counseling is recommended to assess the need for germline testing.
  2. This case has been peer reviewed by two doctors.
  #T-59600_2 #M-82633_2 2 1105 M0000F
  #T-C4560_2 #M-09183_2 0 1105 000000

其它

出院時情況

- 3.改門診治療

出院指示

藥名	劑量	劑量單位	用法	頻次	數量	數量單位	現況	天數	單位
(□)Takepron OD tab 30mg	1	TAB	PO	QDAC	12	TAB	TH	12	天
5mg-Primperan tab	1	TAB	PO	BID	24	TAB	TH	12	天

出院計劃

- 門診日期
  - date on 2021/10/06

主治醫師_2062F 蔣鋒帆_______ / 住院醫師_2336L 洪湊嚴_______

正式摘要(電子簽章：000000226198733)

W52 / W52

doctorRank:B
